# Asymptomatic pink nodules in an African-American woman

**DOI:** 10.1016/j.jdcr.2024.11.026

**Published:** 2024-11-30

**Authors:** Richard Adam, Katie Roster, Kenneth Shulman, Banu Farabi

**Affiliations:** a New York Medical College, Valhalla, New York; b Dermatology Department, NYC Health + Hospital/Metropolitan, New York, New York; c Dermpath Diagnostics, Westchester, New York; d Dermatology Department, NYC Health + Hospital/Coney Island, Brooklyn, New York

**Keywords:** cutaneous Rosai-Dorfman disease, non-Langerhans cell histiocytosis, sinus histiocytosis with massive lymphadenopathy, SOC (skin of color)

## History

**Fig 1:**
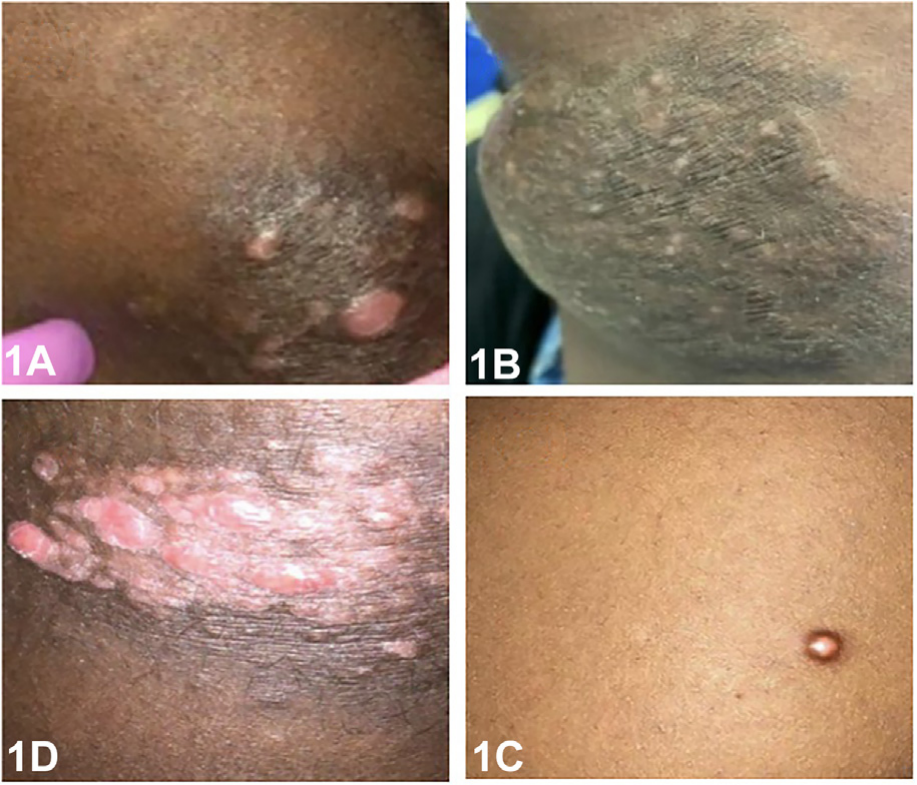

**Fig 2:**
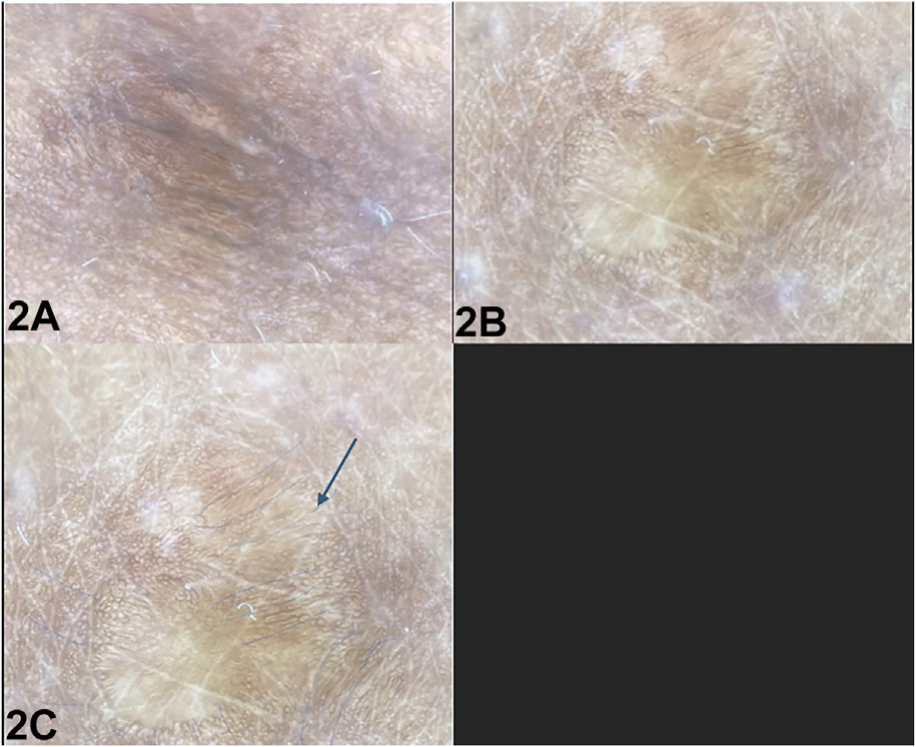

**Fig 3:**
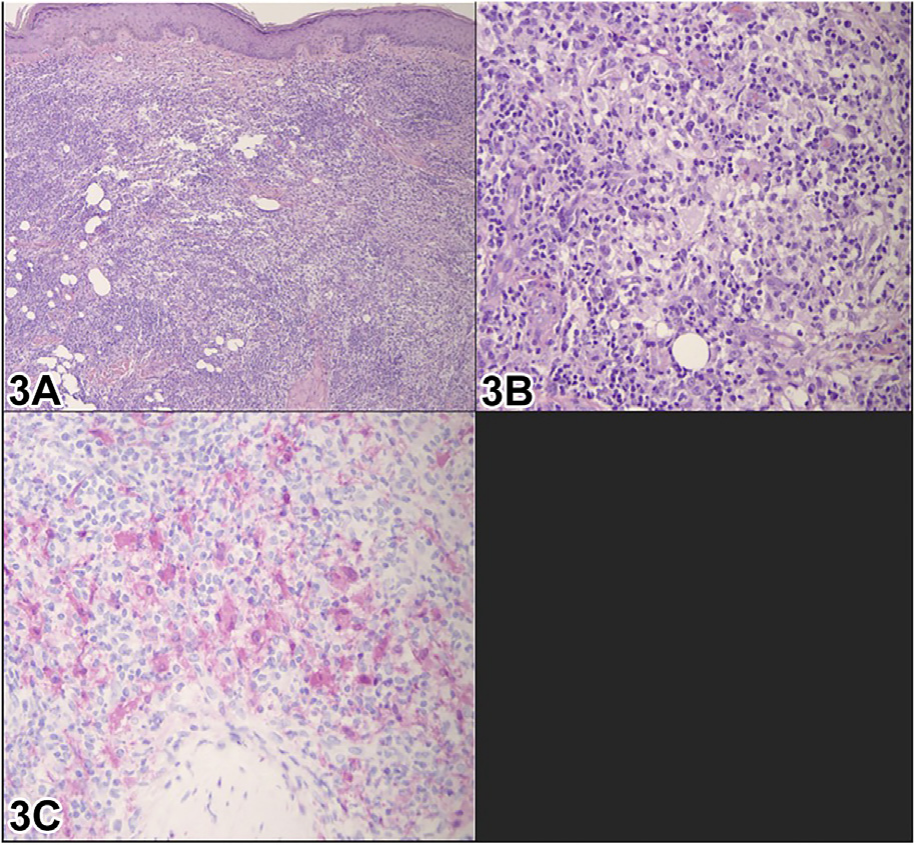

A 28-year-old African-American woman presented with a 6-year history of asymptomatic pink to skin colored nodules on her arms, trunk, upper thighs, and gluteal region with diameters ranging from 5 to 15 cm (Fig 1). Dermatoscopic examination of the lesion of her right thigh revealed a yellow structureless area surrounded by dark brown reticulated lines radiating from the center, without vascular structures (Fig 2). Punch biopsy (Fig 3, *A* and *B*) and immunohistochemical staining (Fig 3, *C*) demonstrated histiocytes positive for S-100 and CD68 but negative for CD1a. Systemic work up (positron emission tomography scan, complete blood count, complete metabolic panel, and lactate dehydrogenase) was negative.

**Question 1: What is the most likely diagnosis based on the history, labs, and images?**

A. Xanthoma disseminatum (XD)
B. IgG4 sclerosing diseases
C. Cutaneous Rosai Dorfman disease (CRDD)
D. Generalized eruptive histiocytosis (GEH)
E. Multicentric reticulohistiocytosis (MRH)

**Answers:**

A. Xanthoma disseminatum (XD) – Incorrect. XD may have a similar cutaneous disease as our patient did. However, XD would typically show Touton giant cells on histology which was not observed. Furthermore, immunohistochemistry for XD would demonstrate CD1a− and S100− negative histiocytes while our case had CD1a- and S100+ histiocytes.
B. IgG4 sclerosing diseases – Incorrect. Even though IgG4 sclerosing disease can present similar cutaneous lesions, cutaneous manifestation is uncommon, occurring in about 4.2% to 6.3% of cases. Histopathology would not show the same extent of histiocytic infiltration as well as the presence of emperipolesis.
C. Cutaneous Rosai Dorfman disease (CRDD) – Correct. With the information given in the case presentation, the underlying etiology that most aligns with this would be CRDD. CRDD is a rare non langerhans cell histiocytosis that most commonly presents with papulonodular skin lesions as seen in our patient. However, clinical features can be heterogeneous. Histopathology typically demonstrates histiocytes exhibiting emperipolesis as well lymphocytic infiltrate with immunohistochemistry demonstrating S100+ and CD1a−. It is differentiated from Rosai Dorfman disease (RDD) and RDD with cutaneous manifestation in that there is no systemic symptomatology such as lymphadenopathy.
D. Generalized eruptive histiocytosis (GEH) – Incorrect. GEH may present with macules with a pink to reddish complexion or as one or more nodules and would most likely not present with emperipolesis on histopathology. Additionally, it would stain negative for S100.
E. Multicentric reticulohistiocytosis (MRH) – Incorrect. MRH may present with red to brown papules/nodules with histopathology. However, MRH would most likely present with other arthritis as well. MRH also demonstrates large epithelioid histiocytes with CD1a+ on histopathology. Emperipolesis is also not characteristic of MRH.

**Question 2: What treatment has shown to be the most efficacious for patients with CRDD?**

A. Systemic corticosteroids
B. Methotrexate
C. Thalidomide
D. Surgical excision
E. Retinoids

**Answers:**

A. Systemic corticosteroids – Incorrect. While corticosteroids have been used to treat CRDD, Dhrif et al performed a systematic review showing that about 30% of patients experienced complete response with systemic and 20% of patients experiencing complete response with local corticosteroids and recommended.^1^
B. Methotrexate – Incorrect. Methotrexate has also been used to treat CRDD, but the literature has demonstrated that about 20% of patients exhibit a complete response and that methotrexate should be considered as a second line for multifocal CRDD.
C. Thalidomide – Incorrect. According to the literature, CRDD has been shown to have a complete response to thalidomide in about 30% of patients.^1^
D. Surgical excision – Correct. Both reviews by Alba et al and Dhrif et al demonstrated that surgical excision was the most efficacious treatment and had the highest complete response rate.^1^^,^^2^ According to Alba et al, initial observation is recommended for asymptomatic RDD, with 20% to 50% of nodal/cutaneous cases showing spontaneous resolution. For symptomatic single-site disease, options include surgical resection, which proved effective in our patient.^3^
E. Retinoids – Incorrect. Retinoids demonstrated a complete response rate in about 10% of patients.^1^

**Question 3: What features have been reported on dermoscopy for patients with CRDD?**

A. Yellow homogenous area surrounded by an erythema background accompanied by smaller vessels
B. Atypical pigment network with a blue white veil and atypical vascular pattern
C. Multiple violaceous lacunae and mild brown pigmentation
D. Red yellow center with a discrete erythematous halo described as a “setting sun”
E. Multiple linear branching vessels overlaying translucent yellowish orange globular structures

**Answers:**

A. Yellow homogenous area surrounded by an erythema background accompanied by smaller vessels – Correct. To our knowledge, there have been 8 reports of yellow homogenous area surrounded by a light red background accompanied by smaller vessels describing CRDD on dermoscopy. Hu et al had also described follicular openings containing yellowish keratitis plugs and white structureless materials on initial diagnosis.^4^ However there is only one report of dermoscopic findings of CRDD in skin of color patients. Rodriguez-Blanco et al described cotton like white surrounded by light red background with no vasculature present.^5^ Our patient similarly did not show any vasculature on dermoscopy. However, we noted gray reticular lines which may be more visible with skin of color patients. These findings contribute to a gap in the literature where dermatoscopic findings are largely under-reported or are not utilized in the diagnosis of CRRD, especially in African American patients. These findings may aid in diagnosis, enabling earlier treatment and resolution.
B. Atypical pigment network with a blue white veil and atypical vascular pattern – Incorrect. These findings on dermoscopy would be most characteristic of melanoma.
C. Multiple violaceous lacunae and mild brown pigmentation – Incorrect. This description would most align with Langerhans cell histiocytosis.
D. Red yellow center with a discrete erythematous halo described as a “setting sun” – Incorrect - These findings would be exhibited in Juvenile xanthogranuloma (JXG).
E. Multiple linear branching vessels overlaying translucent yellowish orange globular structures – Incorrect. This would be most indicative of cutaneous sarcoidosis.

## Conflicts of interest

None disclosed.
